# Potential Coagulation Factor-Driven Pro-Inflammatory Responses in Ovarian Cancer Tissues Associated with Insufficient O_2_ and Plasma Supply

**DOI:** 10.3390/ijms18040809

**Published:** 2017-04-12

**Authors:** Shiro Koizume, Yohei Miyagi

**Affiliations:** Molecular Pathology and Genetics Division, Kanagawa Cancer Center Research Institute, 2-3-2 Nakao, Asahi-ku, Yokohama 241-8515, Japan; miyagi@gancen.asahi.yokohama.jp

**Keywords:** ovarian cancer, hypoxia, coagulation, inflammation

## Abstract

Tissue factor (TF) is a cell surface receptor for coagulation factor VII (fVII). The TF-activated fVII (fVIIa) complex is an essential initiator of the extrinsic blood coagulation process. Interactions between cancer cells and immune cells via coagulation factors and adhesion molecules can promote progression of cancer, including epithelial ovarian cancer (EOC). This process is not necessarily advantageous, as tumor tissues generally undergo hypoxia due to aberrant vasculature, followed by reduced access to plasma components such as coagulation factors. However, hypoxia can activate TF expression. Expression of fVII, intercellular adhesion molecule-1 (ICAM-1), and multiple pro-inflammatory cytokines can be synergistically induced in EOC cells in response to hypoxia along with serum deprivation. Thus, pro-inflammatory responses associated with the TF-fVIIa–ICAM-1 interaction are expected within hypoxic tissues. Tumor tissue consists of multiple components such as stromal cells, interstitial fluid, albumin, and other micro-factors such as proton and metal ions. These factors, together with metabolism reprogramming in response to hypoxia and followed by functional modification of TF, may contribute to coagulation factor-driven inflammatory responses in EOC tissues. The aim of this review was to describe potential coagulation factor-driven inflammatory responses in hypoxic EOC tissues. Arguments were extended to clinical issues targeting this characteristic tumor environment.

## 1. Introduction

Epithelial ovarian cancer (EOC) is a general term representing neoplasms in the pelvic or peritoneal cavity, as the origin of most EOC cases is not the ovary [1]. EOC accounts for approximately 3% of cancers in women and thus represents the most lethal gynecologic malignancy worldwide [2]. EOC is associated with relatively poor prognosis because of the lack of sufficient diagnostic and therapeutic methods. The 5-year survival of this type of cancer is 46.2% [2]. Moreover, there are difficulties in treating this disease when patients have relapsed or are diagnosed at a late stage. EOC can be classified based on multiple histologic [1,3,4], genetic [3], and functional [5] subtypes, such as high- and low-grade serous and clear-cell carcinoma (CCC) [1,3,4,5]. Low-grade serous and CCC subtypes are known to be relatively chemoresistant [3,4,6,7]. Accordingly, greater understanding of the relationship between the heterogeneity of EOC and the underlying biology should result in promising new treatment strategies.

**Figure 1:**
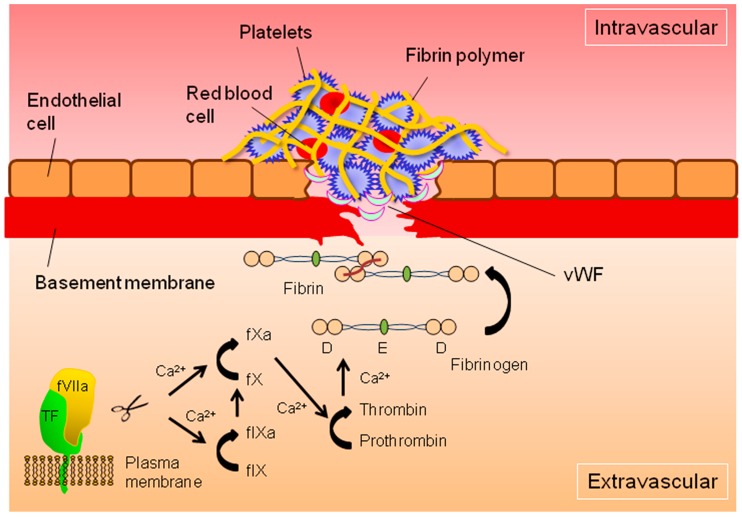
Extrinsic coagulation cascade initiated by tissue factor (TF)-activated coagulation factor VII (fVIIa) dimer formation on the surface of extravascular cells. The TF-fVIIa complex triggers calcium ion-dependent sequential enzymatic reactions in response to injury of blood vessels composed of endothelial cells and the vessel wall. Formation of fibrin polymers together with platelets and red blood cells leads to clot formation to halt bleeding. This fibrin deposition process is supported by von Willebrand factors (vWFs). Blood coagulation completes by clot formation with other factors, such as platelets and red blood cells. Schematics of fibrin(ogen) with characteristic domains D and E are also shown. fXa: activated factor X; flXa: activated fIX; 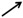: cleavage; 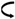: conversion.

Tissue factor (TF) is a transmembrane glycoprotein that is expressed in various normal and cancerous tissues (Figure 1). Blood coagulation factor VII (fVII) is a precursor serine protease produced in the liver and then secreted into the bloodstream. TF functions as a receptor for fVII, and binding initiates the extrinsic blood coagulation cascade, including generation of activated factor X (fXa) via production of the active form of fVII (fVIIa) (Figure 1). The serine protease activity of fVIIa within the TF-fVIIa complex triggers a series of enzymatic reactions, finally leading to clot formation composed of platelets and red blood cells covered with fibrin polymers (Figure 1) [8,9,10]. The fVII pro-coagulant protease, together with fXa, is also responsible for the activation of a cellular signaling cascade by cell surface cleavage of protease-activated receptors (PARs), in normal and cancer cells, including EOC cells [9,10]. In addition to coagulation activity, the cytoplasmic domain of TF also plays critical roles in cancer biology [9,10,11].

Pro-coagulant-driven inflammatory responses play key roles in the progression of various types of cancer [12,13,14,15] including EOC [16,17,18]. The TF-fVIIa complex can contribute to cellular inflammatory responses via direct activation of PAR2 and indirect activation of PAR1 and PAR2 via fXa [11,19] in a coagulation-independent manner, followed by activation of cellular signaling cascades [20]. In addition, fibrin(ogen), an end product of the coagulation cascade (Figure 1), is involved in the inflammatory response in various diseases, including cancer, as this coagulation factor mediates recruitment of immune cells via adhesion molecules [21].

In general, cancer tissues have poorly organized blood [22,23,24] and lymphatic [23,24,25] capillary networks, leading to reductive conditions called hypoxia. Cancer cells have various molecular mechanisms to overcome and adapt to such harsh conditions. The gene encoding TF can be transcriptionally upregulated in response to hypoxia, potentially via the early growth response-1 (EGR-1) transcription factor, in various cancer cells including EOC cells [26,27,28,29,30]. In addition, the gene expression of fVII is inducible in response to hypoxia in EOC cells. However, its regulatory transcriptional mechanisms are distinct from those controlling TF expression [28,29,31,32].

Given that tissue hypoxia is a result of aberrant tissue vascularization, cancer cells in tumors should also undergo starvation of plasma components in addition to O_2_ deficiency. Indeed, recent studies have revealed that the limited supply of plasma lipids, in addition to O_2_, is responsible for the induction of expression of key genes required for compensation of the cellular lipid deficiency in cancer cells [33]. A previous study showed that transcriptional induction of the *FVII* gene in CCC cells under hypoxia is synergistically enhanced when cells are cultured without serum [32]. Moreover, the gene encoding intercellular adhesion molecule-1 (ICAM-1), a cell surface mediator of the immune response, is robustly expressed in CCC cells in response to simultaneous exposure to hypoxia and serum deprivation conditions [34]. This experimental evidence implies that inflammatory responses mediated via the cancer cell-derived TF-fVIIa complex and ICAM-1 play vital roles in EOC progression. However, it is expected that the functions of these proteins are modulated under such harsh conditions given that characteristics of tumor components, such as the extracellular matrix, stromal cells, and tissue interstitial fluid, may also be altered in response to hypoxia. The main aim of this review is to discuss potential coagulation factor-driven pro-inflammatory responses within EOC tissues that are insufficiently supplied with O_2_ and plasma components.

## 2. Potential Relationship between Blood Coagulation and Inflammatory Factors in EOC Tissue: Overview of Current Knowledge

### 2.1. TF-fVIIa-Dependent Phenotypes of EOC Cells

Previous studies demonstrated that the extrinsic but not intrinsic blood coagulation mechanism initiated by the TF-fVII interaction (Figure 1) [8] is closely involved in the biology of cancer cells [9,10,11,19,20], including EOC cells [10]. The extrinsic coagulation cascade consists of sequential enzymatic reactions, finally resulting in fibrin formation (Figure 1). Fibrin monomers are tethered to each other to form homopolymers, leading to clot formation with the assistance of other blood components such as platelets, red blood cells, and von Willebrand factors (vWFs) [8,35] (Figure 1).

Enzymes responsible for this coagulation cascade are known to cause malignant cell phenotypes. TF is overexpressed in CCC cells [10]. Additionally, fVII can be ectopically induced in some EOC cells, including CCC cells, in response to hypoxia via the specificity protein 1 (Sp1)-hypoxia inducible factor-2α (HIF–2α)interaction [28,31,32]. Unlike general transcription mechanisms, this hypoxia-driven transcriptional activation is associated with characteristic epigenetic changes, namely the deacetylation of histones within the promoter region of the *FVII* gene [32].

**Figure 2:**
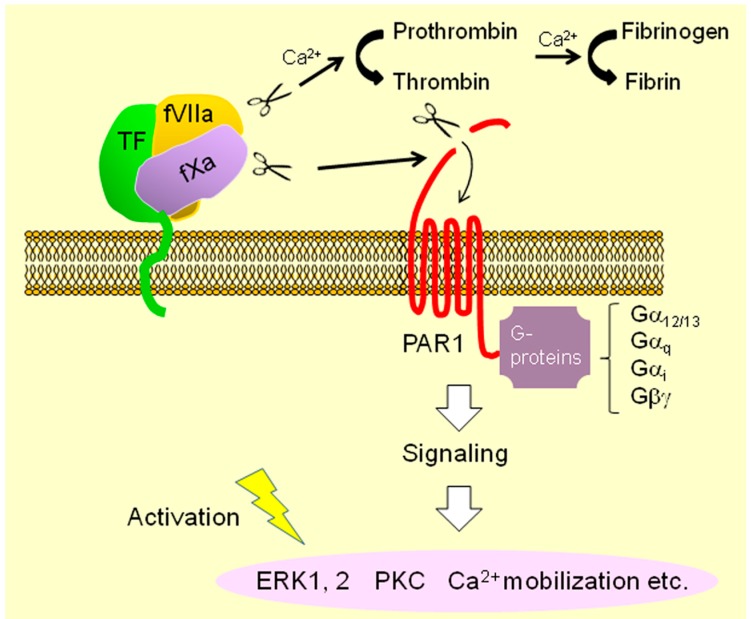
Activation pathways of PAR1 associated with the plasma membrane. In addition to thrombin, the TF-fVIIa-fXa complex can also cleave and activate the G protein-coupled receptor PAR1 to transmit cellular signals. PAR1 can couple multiple G-proteins to activate various signaling mechanisms. PAR: protease-activated receptor; ERK: extracellular signal-regulated kinase; PKC: protein kinase C; 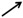: cleavage; 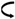: conversion; 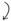: binding; 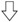: intracellular signaling.

PARs (PAR1–PAR4) are major G protein-coupled receptors that are potentially responsible for transmitting TF-fVIIa-dependent cellular signals [19]. Whether all PARs are involved in the biology of EOC cells is not clear. However, several studies have already investigated the role of PAR1, PAR2, and PAR4 in EOC cell biology [10,29,31,36,37]. Both the motility and invasiveness of CCC cells are increased by ectopic expression of fVII, followed by cell surface TF-fVIIa formation [31]. These phenotypes were considered to be dependent on PAR1, presumably via ectopically synthesized fX, as a ternary TF-fVIIa-fXa complex but not binary TF-fVIIa complex can activate PAR1 [31] (Figure 2). Indeed, recent studies have shown that fX is expressed in CCC cells [29], providing support for this phenomenon. A recent report has also shown that PAR1 facilitates proliferation of non-CCC EOC cells while PAR2 enhances cell motility in an fVIIa-dependent manner [37]. A database search revealed that PAR1 transcript levels are significantly higher in EOC tissues compared with those in normal ovarian tissues [37]. In addition, immune cells treated with TF-fVIIa complex potentially included in ascites can augment secretion of cytokines such as interleukin (IL)-8 (CXCL8), thereby increasing the motility and invasiveness of EOC cells [38]. EOC cells can secrete extracellular vesicles (EVs) associated with high levels of TF or TF-fVIIa associated with procoagulant activity, potentially leading to venous thromboembolism (VTE) [28,29,39]. Shedding of EVs and incorporation of TF into EVs are regulated by the actin-binding protein filamin-A, while only shedding is regulated by PARs [29]. Cell-surface TF-fVIIa activity can be regulated by anti- or pro-coagulants such as anti-thrombin III, phospholipids, and tissue factor pathway inhibitor-1 (TFPI-1) [10]. Intriguingly, a recent report showed that TFPI-2 is highly expressed in CCC cells, suggesting a new biomarker for CCC cells [40,41]. Overall, these findings suggest that components of extrinsic coagulation pathway contribute to aggressive phenotypes of EOC under peritoneal environments.

### 2.2. TF-fVIIa Pathway and Inflammation in Cancer Tissue

The relationship between cancer and inflammation has been widely studied and extensively reviewed [12,13,14,15]. Indeed, several studies have demonstrated that the TF-fVIIa pathway closely correlates with the immune response in cancer tissues [19,20,42,43,44,45,46], as expression of the TF (*F3*) gene can be augmented by pro-inflammatory transcription factors such as NFκB and AP-1 [10,26]. Briefly, TF-fVIIa signaling via PARs augments the production of pro-inflammatory proteins such as tumor necrosis factor-α (TNF-α), interleukins, and adhesion molecules in cancer cells [19]. In contrast, pro-inflammatory factors can enhance transcriptional upregulation of the *F3* gene to increase cellular TF levels [19] via activation of NFκB [26]. The TF-fVIIa complex can directly cleave and activate PAR2 [10,19]. PAR1 is a receptor for thrombin and PAR1 activation requires prothrombin cleavage (Figure 2). However, PAR1 can be cleaved by the TF-fVIIa-fXa complex, as described in the previous section (Figure 2), potentially followed by activation of various signaling cascades including Ca^2+^ mobilization via association with multiple G-proteins (Figure 2) [47]. Also, immune cells are components of the tumor microenvironment (TME) and can cause tumor-promoting inflammation [13]. Previous studies showed that TF-fVIIa signaling in immune cells such as lymphocytes and tumor-associated macrophages (TAMs) contributes to tumor progression [42,43,44,45].

**Figure 3:**
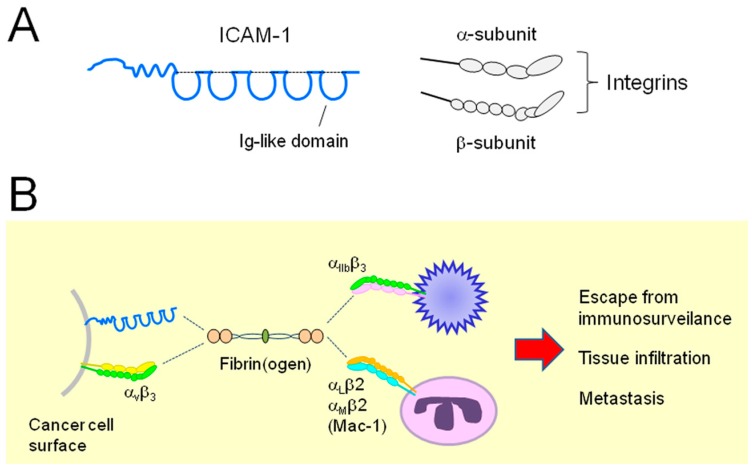
Interaction between receptor molecules on the cancer cell surface and inflammatory cells. (**A**) Schematics of intercellular adhesion molecule-1 (ICAM-1) and integrin structures. Dotted lines in the ICAM-1 structure show disulfide bond formation; (**B**) A schematic of interaction between cancer cells and inflammatory cells, leading to malignancy. ICAM-1 and a heterodimer of integrins on the surface of cancer cells can associate with integrin dimers on the surface of platelets and leukocytes via fibrin(ogen). Dotted lines indicate non-covalent association.

Fibrinogen is composed of two outer D domains tethered with a coiled-coil structure, with an E domain in its center [48] (Figure 1). In addition to its critical role in clot formation (Figure 1), fibrin(ogen) can mediate inflammatory responses in cancer tissues [21]. For example, cancer cells can associate with platelets within the TME. Cancer cells in blood vessels initially need to associate with the vessel wall when they metastasize [49]. In EOC models, studies showed that platelets can augment cell proliferation [50], pro-angiogenic and -survival [51] signaling, and survival under shear stress conditions [52]. Other studies further demonstrated that platelets correlate with EOC tumor growth [49], chemoresistance [51], and thromboembolism [16]. Cancer cells can associate with platelets to enhance their tissue infiltration [49,53]. These cell–cell interactions are known to protect cancer cells from immunosurveillance to promote metastasis [49]. Platelets and leukocytes such as polymorphonuclear leukocytes can associate with cancer cells by association to fibrin to promote metastasis [54,55] (Figure 3). Fibrin(ogen) can be a key regulator of these molecular processes, as cell surface glycoproteins, ICAM-1, and integrins on the cell surface function as receptors of this pro-coagulant [54,56]. These molecular events are expected to influence the pathophysiology of EOC. Indeed, the plasma levels of platelets, lymphocytes, and fibrinogen are significantly associated with the prognosis of EOC patients [57].

### 2.3. Relationship between ICAM-1 and EOC

ICAM-1 is a transmembrane glycoprotein classified in the immunoglobulin superfamily and is expressed in endothelial and immune cells [54,56,58,59]. ICAM-1 consists of five immunoglobulin-like domains (Figure 3A) [56]. ICAM-1 functions in cell–cell and cell–extracellular matrix (ECM) interactions and plays multiple roles in the tissue immune response [56]. Interactions between cell surface ICAM-1 and platelets and leukocytes are mediated by its direct binding to heterodimeric complexes of integrins (LFA-1 and Mac-1) [60,61,62] (Figure 3B). Many studies have shown that various cancer cells, including breast, bladder, pancreatic, and oral cancer cells and cells within the tumor environment, overexpress ICAM-1 [58,63,64,65,66,67,68]. Aberrant expression of this cell surface protein results in elevated cell motility [58,59,60,61,62,63], invasiveness [64], angiogenesis [56], and leukocyte infiltration [69].

Roles of ICAM-1 in EOC cell biology have also been reported. Unlike the effects on cancer cells described above, ICAM-1 expression is inversely correlated with malignancy of EOC cells. ICAM-1 expression is reduced in various EOC cells and tissues compared to normal ovarian surface epithelial cells [70]. Furthermore, forced expression of ICAM-1 inhibited the proliferation of multiple EOC cells [71]. The promoter region of the *ICAM1* gene in some EOC cells is highly methylated at CpG sites, and the expression of *ICAM1* is recovered upon treatment of cells with a DNA demethylating agent [71] or methyltransferase inhibitor [72]. These experimental results suggest that ICAM-1 suppresses the malignancy of EOC cells.

In contrast, immunoassays revealed that ICAM-1 levels are elevated in ascites of EOC patients [73]. Single nucleotide polymorphisms within the *ICAM1* gene are closely associated with risk and prognosis of EOC [74,75], suggesting that ICAM-1 correlates with EOC progression. Platelet adhesion to EOC cells could induce ICAM-1 expression in these cells to promote angiogenesis and cell survival, suggesting that ICAM-1 promotes malignant phenotypes [36]. Indeed, ICAM-1 is inducible in CCC cells under starvation of both O_2_ and long chain fatty acids (LCFAs), thereby facilitating cell survival and tumor growth [34]. Furthermore, a very recent study showed that an analogue of curcumin, a component of the turmeric spice, can suppress EOC progression in association with reduced expression of ICAM-1, presumably by NFκB inhibition [76]. These experimental results suggest that ICAM-1 bidirectionally functions in the progression of EOC.

### 2.4. Relationship between Integrins and EOC

As described above, integrins function in the association of EOC cells to platelets and immune cells via fibrinogen to facilitate tumor progression. Differential dimeric complex formations between α- and β-subunit of integrins are responsible for the diverse cell–cell interactions [77]. The α_II_b-β_3_ complex mediates the interaction between cancer cells and platelets [55], while the α_L_-β_2_ (LFA-1) and α_M_-β_2_ (Mac-1) complexes are responsible for the association of cancer cells to leukocytes [55], enabling EOC cells to exert immune responses. In addition, various studies have shown that interactions between EOC cells with ECM components initiate cellular signaling mechanisms to augment cell migration, invasion, and survival [78], particularly via the α_5_-β_1_ complex [79,80,81].

Cell surface integrins may also contribute to EOC biology by regulation of the TF-fVIIa pathway. Membrane-associated full-length TF and alternatively spliced membrane-free TF (asTF) can bind to multiple integrin dimers to activate different cellular signaling pathways, thereby facilitating breast cancer progression [9]. Furthermore, β1-integrin on the surface of endothelial cells can associate with asTF to augment the expression of adhesion molecules, enabling monocytes to undergo trans-endothelial migration into tumor tissue [82]. These events may be more important for CCC cells compared to other EOC cells, as this histological subtype highly expresses TF and fVII [10,28,29,32]. However, TF–integrin interactions in EOC cell models have not been reported so far.

A previous report showed that statin inhibits CCC cell growth by reduction of the ECM protein osteopontin, which can bind multiple integrin dimers to promote cell invasiveness [83]. Furthermore, integrin-linked kinase, which binds the cytoplasmic domain of integrins and modulates their function, is overexpressed in EOC cells, including CCC cells [84]. These results indicate that integrins can greatly affect the biology of EOC cells.

## 3. Potential TF-fVIIa-Driven Inflammatory Responses within Hypoxic EOC Tissue

The TME consists of a solid phase composed of many stromal cells, such as immune cells and fibroblasts [85], as well as proteins and carbohydrates, such as collagen, glucosaminoglycan, and hyarulonan [25]. The TME is also composed of a fluid phase called the tissue interstitial fluid (TIF), which can be a vehicle of many plasma-derived substances. As described above, the majority of solid tumor tissues are characterized by hypoxic environments. Measurement of hypoxia status by various monitoring and imaging techniques indicated that this is true for EOC tissues [10,86,87,88]. Indeed, investigations of hypoxia in EOC have been increasing [89,90,91].

**Figure 4:**
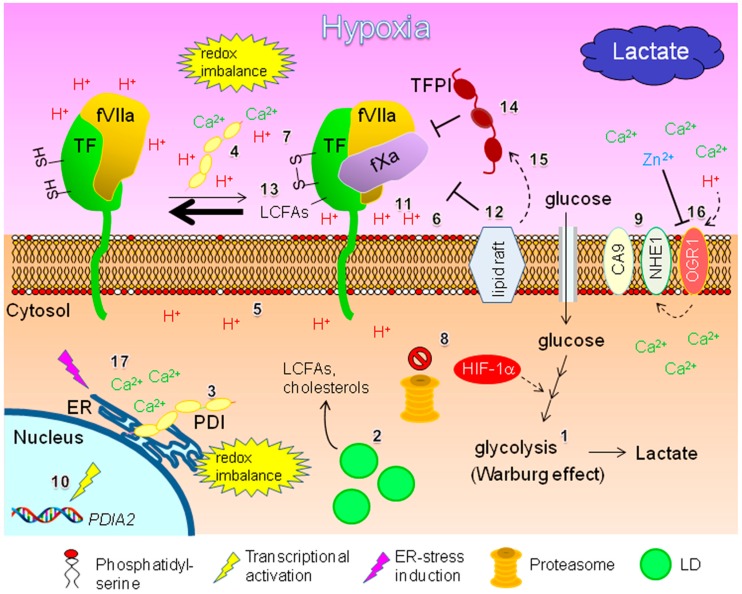
Potential contribution of protein disulfide isomerase (PDI) and various environmental factors in regulation of the TF-fVIIa complex on the surface of epithelial ovarian cancer (EOC) cells. Due to hypoxia, followed by acidification of tumor microenvironment (TME) by lactate production, the cell surface PDI is expected to be inhibited. In this case, TF shifts to its reduced inactive form (designated with the bold black arrow), although encrypted TF may still transmit signals [94]. Additionally, TF-fVIIa activity can be affected by various hypoxia-related cellular and environmental factors such as regulation of the *PDIA2* gene, lipid metabolism, endoplasmic reticulum (ER) stress, proton exchanger molecules, phosphatidylserine, and metal ions. Dashed arrows and T-bars indicate the activation process and suppression process, respectively. LD: lipid droplet; TFPI: tissue factor pathway inhibitor-1; LCFAs: long chain fatty acids; HIF: hypoxia inducible factor. See text for numbers (1–17).

Unlike normal cells, metabolism in cancer cells is mainly regulated by aerobic glycolysis, which is known as the Warburg effect [92] (Figure 4, number 1). This metabolic change accentuates lactate production, especially under hypoxia, leading to an acidic TME. In addition, cellular lipid metabolism can be reprogrammed and lipid anabolism rather than catabolism is predominant with synthesis of lipid droplets (LDs) [33] (Figure 4, number 2). This altered lipogenesis under hypoxia contributes to progression of cancers including EOC [33]. Given the poor supply of plasma lipids, reprogrammed lipid metabolism may affect TF-fVIIa-mediated inflammatory responses in hypoxic EOC tissues. Thus, in this section, potential alterations in TF-fVIIa functions in hypoxic EOC tissues will be presented.

### 3.1. Potential TF Regulation

#### 3.1.1. Regulation by Protein Disulfide Isomerase and Phosphatidylserine: Effect of Hypoxia and Intra-Tumoral pH Level

Protein disulfide isomerase (PDI), with 491 amino acids, contains the endoplasmic reticulum (ER)-retention signal peptide sequence, KDEL [93], and is thus a resident protein of the ER (Figure 4, number 3). PDI is composed of four distinct domains and catalyzes disulfide bond formation between inter- or intra-molecular cysteine residues in an O_2_-dependent manner to regulate physiological protein functions [93]. PDI can also be secreted and associate with the cell surface (Figure 4, number 4) Thus, PDI is also expected to play multiple roles in normal cell function by regulation of the folding process of cell surface proteins. However, the mechanism of this cell surface process is unclear.

The function of TF can be post-translationally regulated by a conformational change, called “encryption-decryption”. This process is regulated by a plasma membrane component, an anionic phospholipid such as phosphatidylserine (PS) [95,96]. Unlike phosphatidylcholine and shingomyelin, PS mainly resides in the inner leaflet of the plasma membrane and under normal conditions cannot be responsible for regulation of TF function (Figure 4, number 5). However, in response to various cellular stimuli associated with Ca^2+^ influx, the asymmetrical PS could be exposed to the extracellular space [97,98], thereby modulating TF function [95,96] (Figure 4, number 6). Much experimental evidence has shown that PDI-driven thiol–disulfide exchange contributes to thrombosis in vivo [97,98] (Figure 4, number 7). Disulfide bond formation between Cys^186^-Cys^209^ residues is critical for the expression of TF function [93] (Figure 4). However, it is still an issue of debate over recent years as to whether cell surface PDI can regulate TF function. A recent review noted that extracellular PS alone is not able to fully activate cell surface TF, but PS and PDI can cooperatively activate cell surface TF [96].

Regarding cancer, many studies have shown that PDI is overexpressed in cancer cells, including EOC cells [99]. In addition, proteome analysis of plasma membrane proteins revealed the presence of PDI on the surface of multiple cancer cells including EOC cells [100]. Thus, PDI on the cell surface may function in some cancer types, potentially via TF-fVIIa complex formation, although the cell surface expression of PDI may be cell type-dependent.

Cancer cells undergo aerobic glycolysis to obtain ATP (Figure 4, number 1) [92]. This metabolism can be enhanced when cells are exposed to hypoxia, as the transcription factor HIF-1α is accumulated in cells due to inhibition of proteasome function (Figure 4, number 8) [33]. The TME tends to become acidic because of the production of lactate during this metabolic process [33]. Cancer cells can regulate intra- or extracellular pH levels by carbonic anhydrase 9 (CA9) and sodium/proton exchanger 1 (NHE1) on the cell surface (Figure 4, number 9). Indeed, CA9 is overexpressed in EOC tissues and its aberrant expression correlates with significant clinical parameters, such as disease prognosis [101,102]. The predominance of NHE1 over CA9 in controlling pH levels within EOC cells is unclear [103]. The pH levels within extracellular and intracellular space are maintained within 6.2~6.9 and 7.1~7.7, respectively, for proper cell functioning [103]. Measurement of pH within the tumor TIF revealed that acidity in the TIF (pH = ~6.9) is indeed higher than that in subcutaneous interstitial fluid (SIF) (pH = ~7.3) [104]. These data are consistent with higher concentrations of lactic acid in the TIF (~12 or ~20 mg/L) compared with those in the SIF (~5 mg/L) [103]. These differences should be distinct if these parameters derived from local hypoxic areas can be compared to those within normal tissues.

Given the O_2_-dependence and optimum pH for PDI functioning, PDI is expected to efficiently function within relatively well-perfused tumor tissues associated with medium pH conditions [105]. Full functioning of PDI is unlikely, given poor O_2_ concentrations and an acidic TME. However, PDI within EOC tumor tissues may still function under low O_2_ concentration, as it can still function at pH values lower than 5 [106]. Additionally, PDI can be upregulated in cancer cells in response to hypoxia [107,108] (Figure 4, number 10). TF function under severe hypoxia may also be impaired by a PS-mediated mechanism. Negative charges on the PS molecule are essential in the TF decryption process, followed by formation of an active TF-fVIIa complex [95,96]. It is expected that the higher concentrations of protons (H^+^) (Figure 4, number 11) under hypoxia neutralize negative charges and then inhibit cell surface PS activity. Collectively, PDI- and PS-driven regulation of cell surface TF under hypoxia may be possible; however, their relative contributions would be context-dependent. Additionally, pharmacological inhibition of PDI suppresses malignant phenotypes, indicating that an anti-PDI strategy is promising for some cancer types [99]. However, whether the inhibition of ER- and/or cell surface-associated PDI is responsible for this anti-cancer effect is unclear.

#### 3.1.2. Lipid-Mediated Regulation

The activity of TF is closely associated with cellular lipids other than PS. Lipid rafts (Figure 4, number 12) are a microdomain of the plasma membrane containing high amounts of cholesterol [95,96]. Cryptic and inactivated TF associates with lipid rafts. Indeed, disruption of lipid rafts by removing cholesterol from the plasma membrane promotes decryption, resulting in active TF. LCFAs (palmitic and stearic acids) are likely to modulate cell surface TF activity (Figure 4, number 13) [95]. In particular, palmitoylation of TF at Cys^245^ directs TF to the membrane lipid rafts. This mechanism may involve phosphorylation of TF, because phosphorylation at the Cys^258^ site reciprocally correlates with the palmitoylation of TF [95,109].

Generally, de novo lipogenesis followed by LD generation (Figure 4, number 2) is accelerated in cancer cells, particularly under hypoxia [33]. This is true for EOC cells [33]. Some reports have shown altered lipid metabolism in EOC cells. Thus, it is likely that cancer-specific lipid metabolism regulates cell surface TF activity. Stored lipids (LCFAs and cholesterols) in LDs can be utilized as needed by lipolysis for energy source and/or materials for membrane synthesis (Figure 4) [33]. Also, expressions of genes responsible for LCFA biosynthesis in EOC cells are enhanced, whereas those associated with de novo cholesterol synthesis are suppressed [110]. A very recent report demonstrated that the *ARID1A* gene mutation, which is closely associated with the malignancy of CCC cells, results in downregulation of the mevalonate pathway responsible for cholesterol biosynthesis [111]. This experimental evidence raises the possibility that TF on the surface of EOC cells may be activated by disruption of lipid rafts from insufficient de novo synthesis of cholesterol. In this case, increased LCFAs may negatively regulate cell surface TF as they can direct TF to lipid rafts.

#### 3.1.3. Potential Regulation by MicroRNAs

MicroRNAs (miRs) are regulatory RNAs that are frequently dysregulated in cancer cells, including EOC cells, and can contribute to cancer progression [112]. Previous reports showed that miR-126 and -223 can post-transcriptionally downregulate TF expression in monocytes [113] and endothelial cells [114], respectively. These miRs can be upregulated in EOC cells [112]. Moreover, other studies demonstrated that hypoxia can decrease expression of these miRs in some non-cancer cells [115,116]. Thus, it is likely that miRs influence the expression level of TF in EOC cells in response to altered supply of O_2_ and plasma components.

#### 3.1.4. Potential Involvement of TFPIs

Tissue factor pathway inhibitor-1 (TFPI-1), also known as TFPI, is a serine protease inhibitor with two isoforms and candidate regulator of cell surface TF-fVIIa [117]. A recent study reported hypoxia-driven suppression of TFPI-1 expression in breast cancer cells [118]. This transcriptional repression can be mediated via an authentic HIF-1α-dependent mechanism [118]. In addition, TFPI-1 downregulation can occur in endothelial cells via HIF-2α [119]. Conversely, TFPI-1 expression was found to induce HIF-1α expression in neuroblastoma cells even under normoxia conditions, thereby enabling cells become drug resistant [120]. It is intriguing that this serine protease inhibitor is also expressed in EOC cells and plays multiple roles in the regulation of TF-fVIIa activity. A previous study showed that EOC cells express TFPI-1 and the expression levels did not differ between histological subtypes [121].

TFPI-2, a protease inhibitor with weaker activity against TF-fVIIa complex than TFPI-1, is more constitutively and highly expressed in CCC cells compared to other EOC cells [40,41]. Whether TFPI-2 can be upregulated in response to hypoxia in EOC cells and contribute to regulation of cell surface TF function remains unclear. However, one study showed that the transcript level of TFPI-2 is increased in a von Hippel–Lindau (*VHL*) gene disruption-dependent manner in renal cancer cells [122], suggesting that HIFs may contribute to this transcriptional activation. Indeed, the *VHL* gene is frequently lost in EOC, especially in CCC cells, supporting a potential HIF-driven TFPI-2 expression [122,123,124]. Overall, a hypoxia-driven induction of TFPIs in EOC cells has not been reported yet. However, it is likely, given characteristics of EOC tissues such as hypoxia and *VHL* dysfunction in EOC cells (Figure 4, number 14).

TFPI-1 activity seems to be influenced by lipid rafts in the plasma membrane. As described above, TF-fVIIa activity can be repressed by binding to lipid rafts. TF-fVIIa may be activated once lipid rafts are disrupted by altered lipid composition, such as decreased cholesterol level. One study showed that TFPI-1 activity on the surface of Chinese hamster ovary cells could be enhanced by its binding to caveolae, a kind of lipid raft [125] (Figure 4, number 15). This suggests that TFPI-1 can indirectly suppress TF-fVIIa activity by lipid rafts. In contrast, another report showed that the ability of TFPI-1 to suppress the TF-fVIIa complex is independent of lipid rafts [126]. Together, this suggests the possibility that TF-fVIIa activity on the EOC cell surface is affected by multiple TFPI-driven mechanisms.

#### 3.1.5. Possible Involvement of Integrins

As described above, integrin α5 complexed with β1 subunit plays key roles in the biology of EOC cells. To date, various studies have shown that this integrin subunit is inducible in various non-EOC cancer cells in response to hypoxia, non-hypoxia HIF expression conditions, and Sp1-dependent mechanisms [127,128,129]. In addition, coagulation factors can function as ligands for integrins [130]. The α5-β1 subunit can interact with multiple coagulation factors such as fX, fibrinogen, and vWF [130]. Thus, the α5 subunit is expected to largely influence TF-fVIIa-dependent inflammatory responses if hypoxia-driven expression of the α5 subunit is possible in EOC cells. One study in an endothelial cell line showed that the cell surface full-length form of TF can bind integrin dimers, especially those composed of α3 and α6 subunits, but could not bind the α5 subunit [131]. The precise nature of the physical interaction between TF and the α5 subunit in EOC cells is currently unclear. Thus, the relationship between hypoxia-driven expression of integrins and TF-fVIIa function in EOC cells remains obscure. However, EOC cells could aberrantly produce trypsin [132], thereby potentially resulting in PAR2-dependent activation of the cell surface α5-β1 complex [133]. Thus, TF-fVIIa possibly augments activity of this integrin complex via PAR2 on the surface of EOC cells.

#### 3.1.6. Potential Involvement of Matriptase and Metalloproteinases

Matriptase (MTP) is a member of the type II transmembrane serine protease family. Recently, this protease, in addition to PAR2, was found to be cleaved and activated by the TF-fVIIa(-fXa) complex to transmit cellular signals [134]. TF on the cancer cell surface within tumors can contact fVII via TIF and/or its ectopic synthesis in cancer cells. This raises the possibility that MTP-mediated cellular signaling reactions can cause malignant phenotypes of EOC cells if cancer cells produce sufficient levels of MTP. Indeed, MTP is highly expressed in EOC cells and tissues, and this aberrant expression correlates with disease prognosis [135,136,137,138]. In vitro experiments showed that MTP contributes to the facilitation of motility and invasiveness of EOC cells [138]. The further significance of MTP in EOC biology and its potency as a therapeutic target await future investigation.

Metalloproteinases, such as matrix metalloproteinases (MMPs) and a disintegrin and metalloproteinase with thrombospondin motifs (ADAMTSs), are other candidate mediators of the TF-fVIIa pathway. MMP-2 and MMP-9 can be upregulated in small cell lung cancer cells in a TF-dependent manner [139]. Thus, the same transcriptional activation pathways may be involved in the biology of EOC cells exposed to hypoxia. Indeed, previous studies reported that MMP-2 and MMP-9 are highly expressed in ovarian tumor tissues [140]. These MMPs potentially augment immune responses via activation of PAR1 [141,142] through enhanced secretion by thrombin [143]. Moreover, expression of these MMPs may be augmented in EOC cells in response to hypoxia as increased HIF-1α [144] and Sp1 [145] can target *MMP2* and *MMP9* genes.

ADAMTSs are extracellular proteases responsible for diverse physiological functions such as inflammation and vascular biology [146]. ADAMTS13 cleaves the multimeric vWF precursor to exert a proper blood coagulation process [146]. In addition, TFPI-2 is a binding partner and substrate of ADAMTS1 [147]. ADAMTSs were found to be upregulated in EOC tissues [148]. Thus, it is feasible that ADAMTS as well as MMPs influence coagulation factor-driven inflammatory responses in EOC tissue.

### 3.2. Implications Regarding ICAM-1

#### 3.2.1. Potential Inflammatory Responses under Deprivation of Both O_2_ and Serum

**Figure 5:**
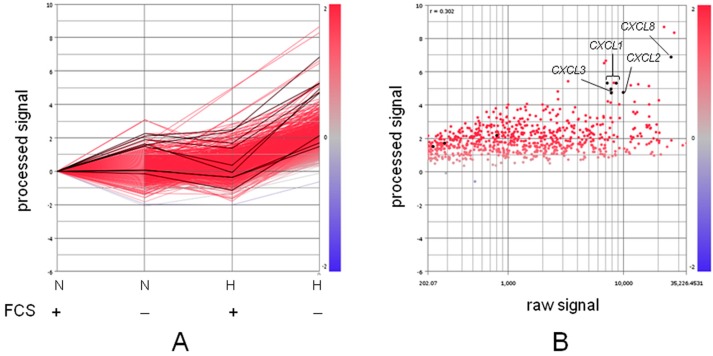
cDNA microarray analysis [34] revealed that interleukins (CXCLs) are upregulated at the highest levels in OVSAYO cells when cells are simultaneously exposed to hypoxia and serum starvation conditions. Cells were cultured under normoxia (N) and hypoxia (H) for 16 h in the presence (+) or absence (‒) of fetal calf serum (FCS), and then total RNA was isolated for transcriptome analysis. (**A**) Line graph representation of gene expression levels under four different culture conditions. Genes synergistically and most activated under “H/FCS‒” condition (688 total measurements) are shown as red lines. Among them, nine genes correspond to CXCLs, as highlighted in black. (**B**) Scatter plot representation of genes shown in (**A**). Expression levels (processed signal) under “H/FCS‒” conditions were plotted as a function of raw signal according to the heat map on the right. Highly expressed CXCLs were assigned to each dot.

As described above, we showed that expression of ICAM-1 is synergistically and strongly enhanced in CCC cells when cells are exposed to hypoxia along with LCFA starvation via interplay among transcription factors Sp1, HIFs, and NFκB. Thus, ICAM-1-mediated inflammatory responses are expected under this severe TME condition. Additionally, our complementary DNA (cDNA) microarray analysis using OVSAYO cells revealed that many genes are synergistically activated in response to simultaneous deprivation of O_2_ and serum encoded pro-inflammatory factors such as many interleukins (ILs, also called as CXCLs) [34,149] (Figure 5) and TNF-α [34,149]. These factors, together with vascular endothelial growth factor (VEGF) secreted from CCC cells exposed to hypoxia likely enhance the permeability of blood vessels, thereby promoting extravasation of coagulation factors and pro-inflammatory cells to augment ICAM-1-mediated inflammatory responses. However, a hypoxic environment is not necessarily favorable for the ICAM-1 mediated response, as vessel permeabilization factors released from cancer cells may also lead to generation of aberrant vasculature. This vascular abnormality may result in inefficient perfusion, followed by inhibition of proper TIF flow.

In addition, lymph angiogenesis and/or lymphatic vessel dysfunction are likely to affect plasma-derived protein levels within the tumor TIF. Indeed, a previous study demonstrated that protein concentrations in the tumor TIF are higher than those in normal tissues [104]. Furthermore, due to the vascular dysfunction described above and increased solid stress associated with the high growth rate of tumors [150], the TIF pressure is higher in cancer tissues than in normal tissues [104,150]. This suggests that dissemination of plasma- and lymph-derived proteins and pro-inflammatory cells within cancer tissues is restricted. In addition, tissue dissemination of plasma-derived molecules and cells is likely to be an important determinant for successful ICAM-1-driven inflammatory responses [104]. Molecular weight, lipophilicity, hydration, and charge of components of ECM and TIF [104,151] are likely critical factors for the dissemination of plasma-derived factors.

Overall, hypoxia along with LCFA starvation can dramatically induce ICAM-1 expression, potentially driving CCC cells to exert inflammatory responses via coagulation factors such as fibrin(ogen). However, it is currently difficult to identify which tumor area is exposed to both hypoxia and limited supply of plasma lipids, as this process would be dependent on a number of factors.

#### 3.2.2. Relationship to Intra-Tumoral Albumin Level

In the advanced stage of EOC, the disease tends to associate with cachexia, leading to poor nutrient status of patients. Malnutrition of cancer patients including EOC patients is common and is related to poor survival. Thus, cachexia is regarded as an important prognostic factor for EOC [152,153]. Cachexia can be estimated by measuring plasma albumin levels [152,153], indicating that plasma albumin concentration can significantly vary depending on the progression of EOC. Hypoxia associated with an insufficient supply of serum lipid (LCFA–albumin complex) causes synergistic activation of the *ICAM1* gene to promote CCC cell survival activity [34]. Thus, we surmised that there must be hypoxic tumor areas particularly associated with low albumin levels. However, how and where such characteristic tumor regions can be generated is unclear. Thus, it is worth discussing here how albumin functions within ovarian tumor environments.

Albumin can be considerably present in human peripheral afferent lymph, and its concentration in lymph is approximately 40% of that in plasma [154]. Component analysis of TIF isolated from EOC tissue and normal ovarian tissue revealed that albumin concentrations are higher in EOC TIF than those of healthy ovary tissues associated with reduced collagen levels [155]. This result was unexpected because investigators initially anticipated that steric exclusion and the negative charge effect due to increased collagen levels in ECM should restrict the available distribution volume, leading to decreased albumin levels within EOC tumor tissues [155]. This phenomenon may be largely due to increased hydration measured on the basis of Na^+^ concentration. Enhanced hydration within EOC tumors compared to that in healthy ovarian tissues increases the available distribution volume, allowing efficient dissemination of albumin [155]. In conclusion, the absolute distribution volume of albumin within EOC tissues can vary depending on density of ECM, degree of hydration, and ionization of ECM components. Thus, the ECM structure may be a crucial determinant of tissue albumin levels. ECM components can vary depending on histological subtype of EOC [155]. For example, a previous study reported that stromal tissues of CCC patients are highly hyalinized, with increased basement membrane materials such as laminin and type IV collagen [156].

Molecular size-dependent exclusion is likely a major determinant of TIF components. Several studies reported equal diffusion of relatively low molecular weight dextran (10~40 kDa) within rat fibrosarcoma, whereas diffusion clearly decreases as molecular weight increases in the range between 40~70 kDa [155,157]. Diffusion of albumin within EOC tissues may follow this principle, as the molecular size of albumin (66 kDa) is within the higher range. In addition, albumin molecules should be neutralized or positively charged due to tissue acidification as it becomes distant from the capillary. Collectively, these arguments imply that tissue dissemination of TIF components is not equivalent between low molecular weight compounds such as glucose and higher molecular weight compounds such as albumin.

Overall, tissue albumin levels vary depending not only on the liquid phase of the TME, such as the hydration and acidity of TIF, but also on characteristics of TIF components, such as molecular size, steric structure, charge, and lipophilicity. Diverse interactions among these factors likely reveal a complex picture regarding distribution of TIF components within aberrantly vascularized EOC tissues. Finally, albumin availability for cancer tissues can be altered depending on individual EOC patients, as it could be associated with different plasma albumin levels.

## 4. Potential Roles of Metal Ions in Coagulation Factor-Driven Inflammatory Responses

### 4.1. Potential Involvement of Zinc Ion

Zinc is an abundant metal in the human body and is essential for physiological functions [158]. A number of proteins require association with the zinc ion (Zn^2+^) to properly regulate critical functions, such as DNA damage response and erasure of harmful reactive oxygen species [158]. Zn^2+^ is vital for innate and adaptive immunities [158,159,160,161]. Zn^2+^ homeostasis is often dysregulated in cancer patients [160,161]. Cancer patients with diminished Zn^2+^ levels suffer from impaired immune function [158]. Indeed, some evidence suggested that Zn^2+^ metabolism within tumor tissues is dysregulated, and Zn^2+^ levels often become lower in plasma and scalp hair of cancer patients compared to normal healthy individuals [159]. Indeed, Zn^2+^ availability in cancer patients could be reduced because albumin, a major vehicle of plasma Zn^2+^, is decreased in association with cachexia [160,161]. Additionally, Zn^2+^ levels are higher in healthy human serum than that of lung cancer patients [161]. However, Zn^2+^ levels become higher in breast and lung cancer tissues although plasma Zn^2+^ levels are reduced [158]. Thus, Zn^2+^ homeostasis can vary depending on cancer types and altered tumor Zn^2+^ levels likely influence coagulation factor-dependent immune responses in EOC tissues.

The ubiquitously expressed transcription factor Sp1 contains a three finger-like structure complexed with Zn^2+^, called a zinc-finger motif (ZFM). The ZFMs at the C-terminal of Sp1 protein facilitate its binding to consensus DNA binding sites [162]. Zn^2+^ is thus essential for Sp1-driven regulation of many genes including most housekeeping genes. Sp1 is also responsible for basal and/or hypoxia-driven expression of TF, fVII, and ICAM-1 genes [32,34]. Moreover, Zn^2+^ is also essential for proper functioning of MMPs [163]. Thus, TF-fVIIa-dependent inflammatory responses may be enhanced if Zn^2+^ levels within EOC tissues are increased. EOC cells can express zinc transporters such as LIV1 [164,165] and hZIP1 [166] to uptake extracellular Zn^2+^, potentially leading to accumulation of Zn^2+^ within cells. However, one study reported that Zn^2+^ levels within EOC tissues and plasma of EOC patients were lower than those in benign tissues and normal controls, respectively [167]. Furthermore, zinc treatment of cancer cells can activate the cell death process, resulting in apoptosis or necrosis [167]. Thus, lower Zn^2+^ levels within EOC tissues are not necessarily unfavorable for the proper function of Sp1.

Taken together, dysregulation of zinc homeostasis within EOC cells is likely to affect coagulation factor-dependent inflammatory responses. The Sp1 transcription factor can be a major candidate regulator of this process through its ZNF motifs. However, knowledge regarding zinc metabolism in EOC cells is currently limited and awaits future exploration.

### 4.2. Potential Involvement of Calcium Ion

In addition to Zn^2+^, H^+^ and calcium ion (Ca^2+^) are key components of the TIF [168]. These factors may be more influential within hypoxic tumor tissues, as cancer cells tend to produce large amount of H^+^ with lactate [33]. This is a vital issue for TF-fVIIa-dependent inflammatory responses given that Ca^2+^ is essential for successful progression of the coagulation cascade and activation of cell surface receptors (Figure 1 and Figure 2). The calcium ion is also essential for the interaction between integrins and coagulation factors [130] and function of MMPs [163]. Furthermore, as already discussed in this review, Ca^2+^ influx contributes to the activation of cell surface TF. PAR1 activation can contribute to cellular Ca^2+^ mobilization (Figure 2). Also, PAR2 activation can increase intracellular Ca^2+^ levels to transmit downstream signaling [19]. Several studies demonstrated that the tissue availability of Ca^2+^ is closely and inversely associated with extracellular H^+^ concentrations [168,169,170]. This reciprocal regulation of cellular H^+^ and Ca^2+^ levels can be mediated by the G protein-coupled receptors ovarian cancer gene receptor 1 (OGR1) (Figure 4) and calcium-sensing receptor (CaSR), respectively [168]. In addition, extracellular H^+^ can activate Ca^2+^ channels [169,170]. OGR1, a cell surface H^+^ receptor ubiquitously expressed in human tissues [170], was initially identified from an EOC cell line (Hey) [171]. OGR1 acts as a sensor of extracellular pH (Figure 4, number 16) [168,169]. OGR1 functions in a pH-sensitive manner and is active around pH 6, a commonly observed extracellular acidity within tumor tissues [170]. Thus, it is expected that OGR1 contributes to acidification of the TME of EOC tissues. Activation of OGR1 in cancer cells by H^+^ exposure can augment NHE1 activity [169] (Figure 4) with increased intracellular Ca^2+^ and inositol triphosphate levels, resulting in facilitation of proton extrusion. Importantly, Zn^2+^ is a known inhibitor of OGR1 (Figure 4) [169].

The ER is an important Ca^2+^ storage organelle [172,173]. ER stress, followed by the unfolded protein response, is a cellular response mechanism associated with incorrect folding of newly synthesized proteins, leading to survival under severe conditions. Hypoxia causes ER stress, thereby releasing Ca^2+^ from the ER (Figure 4, number 17) [172,173]. Ca^2+^ elicits various signaling mechanisms to exert cellular stress responses [168,169,170,171,172,173]. Thus, this ER-mediated regulation of intracellular Ca^2+^ levels likely contributes to extracellular acidification (Figure 4). Hypoxia-driven ER stress can invoke redox imbalance, as O_2_ is required for the catalytic reaction by PDI [33] (Figure 4). In addition, a recent study demonstrated that Ca^2+^ depletion from the ER affects the mobility of ER luminal PDI, also resulting in redox imbalance (reductive shift) of disulfide isomerization in the ER (Figure 4) [174]. This is possibly applicable to cell surface PDI. Collectively, extracellular Ca^2+^ homeostasis within hypoxic EOC tissues could be regulated in association with intra- and extracellular acidity because Ca^2+^ receptors and channels can be intimately linked to H^+^ sensors on the surface of cancer cells. This process is likely influenced by cachexia, as a considerable amount of Ca^2+^ in the blood associates with albumin [175].

## 5. Potential Coagulation Factor-Driven Immune Responses within EOC Tissues Insufficiently Supplied with O_2_ and Plasma Components

**Figure 6:**
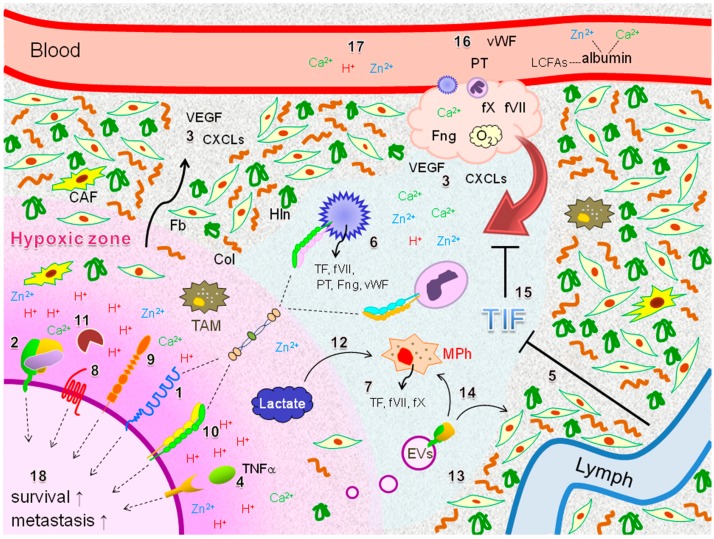
Potential coagulation factor-driven inflammatory responses within TME of EOC exposed to hypoxia. The coagulation factors derived from circulation, stromal cells, and cancer cells can connect EOC cells to platelets and leukocytes. Cell surface receptors and protease can also be activated under hypoxia, potentially transmitting cellular signaling. These molecular events are expected to facilitate survival and metastatic potential of EOC cells. Fb: fibroblast; CAF: cancer-associated fibroblast; TAM: tumor-associated macrophage; Mph: macrophage; Col: collagen; Hln: hyaluronan; PT: prothrombin: Fng; fibrinogen; VEGF: vascular endothelial growth factor; TIF: tissue interstitial fluid; EVs: extracellular vesicles; TNF-α: tumor necrosis factor-α. Dashed lines are indicative of non-covalent association. T-bars indicate suppression process. 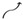: secretion; 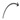: interaction; 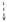: intracellular signaling.

Solid tumor tissues, including EOC tissues, are composed of many factors other than cancer cells, such as various immune cells, fibroblasts, cancer-associated fibroblasts (Figure 6) and tumor-associated macrophages (TAMs) (Figure 6), to generate the characteristic TME structure beneficial for cancer progression [85,156,176,177,178]. ICAM-1 may play primary roles in the immune response in CCC tissue exposed to severe hypoxia (Figure 6, number 1). Furthermore, hypoxia can induce expression of TF and VEGF at the transcriptional level in EOC cells (Figure 6, numbers 2 and 3). TF induction under hypoxia can be dependent on VEGF signaling [179] but is not a direct target of HIF [10]. VEGF induction can be dependent on both HIF-1α and TF signaling [180]. Furthermore, fVII, CXCLs (Figure 5 and Figure 6, number 2 and 3), and TNFα (Figure 6, number 4) are also induced in response to hypoxia, particularly when EOC cells are simultaneously exposed to serum starvation, as in the case of *ICAM1* gene [34]. Increased secretion of VEGF, cytokines and chemokines by cancer cells are indicative of enhanced permeability of capillaries and prompt penetration of plasma coagulation factors into tumor tissues (Figure 6). Furthermore, given the possible dysfunction of the lymph system, followed by failed migration of stromal factors into lymph capillaries (Figure 6, number 5) [104], tumor tissues are potentially pro-coagulant-rich conditions compared to normal tissues. It is further expected that the TF-fVIIa complex is increased on the surface of EOC cells (Figure 6, number 2), resulting in increased fibrin levels.

Previous immunohistochemical experiments have implicated hypercoagulability within EOC tissues, as evidenced by the presence of TF, fV, fVII, and fibrin deposition [181]. Later studies with a specific antistatin probe revealed that fX is expressed in various cancer tissues [182]. Indeed, we recently found that fX presents in CCC cells [29]. Furthermore, this ectopic expression is increased in response to hypoxia [29]. Activated platelets can secrete prothrombin, fibrinogen, and vWF [183] (Figure 6, number 6). Also, macrophages can synthesize TF [184], fVII [45], and fX [182] (Figure 6, number 7). TF synthesis in macrophages can be facilitated in response to ATP exposure via P2X7 receptor signaling in mice. [185]. This inflammatory process can be regulated by PDI to generate TF-positive EVs, thereby leading to thrombosis [185]. These plasma-independent pro-coagulants potentially enhance TF-fVIIa-driven inflammatory responses via PARs (Figure 6, number 8), MTP (Figure 6, number 9), integrins (Figure 6, number 10), and MMPs (Figure 6, number 11). A recent study demonstrated that tumor-derived lactate can augment the expression of pro-angiogenic genes in macrophages [186], suggesting that macrophage-derived pro-coagulants may also be influenced by EOC cell-derived lactate (Figure 6, number 12). Some studies have shown that non-hepatocytic cancer cells can autonomously synthesize fibrinogen [187,188], although ectopic synthesis of prothrombin in cancer cells has not been demonstrated. These results suggest that multiple coagulation factors can be ectopically synthesized within tumor tissue.

Currently, there are no reports on hypoxia-driven expression of PAR1 and MTP, although PAR2 was shown to be increased in response to hypoxia in glioma cells [189]. In addition, no reports have examined hypoxia-driven expression of integrins in EOC cells. The TF-fVIIa complex can be secreted into the TIF as a component of EVs (Figure 6, number 13) [28,29]. It is possible that this extracellular TF-fVIIa may interact with stromal cells and transmit intracellular signals (Figure 6, number 14), as demonstrated in a glioma model [189], to contribute to the aggressiveness of EOC.

Pro-inflammatory proteins within the TME are unlikely to fully function given the high concentrations of H^+^. In addition, higher TIF pressure within tumor tissues may block efficient penetration of plasma components into tumor tissues (Figure 6, number 15). Prothrombin, fibrinogen, vWF, and albumin-LCFA complex are expected to extravasate into tumor tissues (Figure 6, number 16). These factors are expected to differentially disseminate within tissue, as they vary in molecular size [8]. Also, their dissemination can be modulated in relation to histological diversity, tissue components, and physicochemical characteristics of EOC tumors.

In addition, intra-tumoral concentrations of H^+^, Zn^2+^, and Ca^2+^ should be determined depending on the balance of their influx and efflux via cell surface transporter molecules (Figure 4 and Figure 6, numbers 16 and 17), reprogrammed metabolism, and release from cellular ion stores. Also, Zn^2+^ and Ca^2+^ homeostasis can be affected by reduced plasma albumin levels due to cachexia (Figure 6, number 16). A previous study also revealed that protein levels of CA-125, VEGF, and osteopontin in the TIF vary depending on histological subtypes and disease stages of EOC [190]. Therefore, successful recruitment of platelets and leukocytes to EOC cells (Figure 6) can depend on the diverse pattern of interaction between tumor constituents. Finally, in addition to this cell-to-cell contact, survival [10] and metastatic potential [10] of EOC cells may be enhanced due to intracellular signaling via the activation of proteases [10], receptors [34], and adhesion molecules [191] on the cell surface (Figure 6, number 18).

## 6. Clinical Implications

Accumulating experimental evidence has suggested that an anti-coagulant strategy is promising for ovarian cancer patients in addition to generally applied therapeutics. Indeed, use of the recently developed anticoagulation agents, generally called direct oral anticoagulants (DOACs), may be promising, as these drugs that target fXa and thrombin can suppress inflammatory responses [192,193]. However, this strategy is currently applied only to cancer patients who develop VTE [194]. The present review suggests that the EOC tumor region associated with insufficient supply of O_2_ and albumin-LCFA complex potentially tends to undergo coagulation factor-dependent inflammatory reactions. Identification of such tissue areas is expected to be beneficial, as it could generate promising prognosis markers and therapeutic targets. Recent advances in spectroscopic methodology have enabled real-time monitoring of hypoxia regions within tumors [195]. For example, nitroimidazole compound treatment of tumor-bearing mice followed by positron emission tomography (PET) revealed severely hypoxic tumor regions [196]. Specific molecules including proteins, lipids, and lactate within EOC tumors can be directly detected using magnetic resonance spectroscopy (MRS) [197,198]. Furthermore, extracellular acidity can be measured using similar spectroscopy techniques [198]. Therefore, these less invasive detections by PET and MRS may enable us to identify TME associated with both hypoxia and insufficient lipid supply; however, additional methodological improvements are likely required in future studies to detect such small tissue areas. Targeting such specific tumor regions is likely to improve EOC therapy by reducing toxic side effects. However, this strategy may also be disadvantageous for drug dissemination due to the dysregulated vasculature and increased TIF pressure [151]. Future development of highly permeable hypoxia-activated prodrugs such as nitroimidazole derivatives [199] may overcome this problem. In addition, hypoxic tumors are known to be resistant to radiation therapy [200]. Currently, heavy particle beam therapy is considered beneficial to circumvent the radioresistance of hypoxic tumors [201].

Low level of plasma albumin is associated with cachexia, a malnutrition status. Cancer patients with cachexia are known to show poor prognosis. The data described in this review suggest that poor plasma albumin levels may facilitate synergistic induction of multiple genes in hypoxic EOC cells, leading to disease progression. In addition, the albumin-bound paclitaxel preparation (nab-paclitaxel, Abraxane^®^), at 130 nm in size, is promising for EOC therapy [202]. Thus, the efficacy of nab-paclitaxel in EOC should be impaired if the tissue availability of albumin is restricted due to poor vascularization. On the other hand, another study reported that nab-paclitaxel treatment combined with anti-angiogenesis therapy improved tumor blood perfusion of non-small cell lung cancer [203], suggesting that nab-paclitaxel may contribute to tumor vascularization, followed by an improvement of the oxygenation status of EOC tissue. Together this suggests that low albumin levels in cancer patients may cause a high mortality rate not only by malnutrition but also via augmented malignant tumor phenotypes and inefficient drug delivery.

In addition to the anti-coagulation strategy, a therapeutic potential strategy targeting Sp1 may be possible, as this transcription factor plays key roles in the induction of pro-coagulants in response to hypoxia. To date, multiple chemical inhibitors of Sp1 function are available, such as mithramycins, curcumin, and doxorubicin [204]. However, care should be taken for unwanted side effects, as Sp1 is critical for many normal cell functions and the known Sp1 inhibitors lack target specificity.

## 7. Summary and Perspectives

This review compiled potential coagulation factor-dependent pro-inflammatory reactions within EOC tissues exposed to deficiency of O_2_ and plasma components. Cellular expression levels of proteins discussed in this review such as coagulation factors and ICAM-1 would differ depending on cancer cell types and tumor components. Thus, it is unlikely that this pro-inflammatory response occurs within the TME of all cancer types. Overall, it is possible that the TF-fVIIa complex and ICAM-1 are significantly involved in this mechanism under these harsh conditions. To date, in vitro experiments have shown that the expressions of fVII and ICAM-1 were synergistically increased when CCC cells are simultaneously exposed to hypoxia and serum starvation conditions. Specifically, the albumin-LCFAs complex was identified as a serum component responsible for the *ICAM1* induction. Serum factors involved in *FVII* expression have not been reported. Thus, tumor tissue associated with deficiency of both O_2_ and LCFAs may frequently undergo an inflammatory response associated with coagulation factors and ICAM-1. However, this process is not necessarily advantageous for EOC progression as the supply of plasma factors should be limited. The diffusion of plasma coagulation factors within the TIF of EOC is likely diverse due to the differential composition of ECM components, stromal cells, tissue hydration, and TIF pressure. Physical characteristics of plasma factors, such as charge, steric effect, and molecular mass, are also critical for their distribution within tumors. In addition, it has been reported that in addition to cancer cells, non-cancerous tumor constituent cells also potentially secrete coagulation factors. Thus, these ectopically synthesized pro-coagulants may compensate for insufficient plasma-derived coagulation factors to exert TF-fVIIa-driven inflammatory responses.

Tumor tissues exposed to hypoxia are generally accompanied by extracellular acidification and reprogramming of cell metabolism, thereby modulating TF-fVIIa function via PDI and cellular lipids. These mechanisms may be influenced via the influx–efflux of metal ions. Furthermore, albumin levels within EOC tissues can be altered in association with cachexia, potentially affecting the hypoxia-driven gene expressions required for coagulation factor-driven inflammation reactions. That is, this review revealed that TF-fVIIa-driven pro-inflammatory responses within EOC tissues can be affected by multiple cellular and environmental factors in association with characteristic vasculatures, thereby augmenting malignancy. Future studies will lead to greater understanding of the aggressiveness of EOC, followed by the generation of promising clinical applications.
